# Shorter telomere length increases the risk of lymphocyte immunodeficiency: A Mendelian randomization study

**DOI:** 10.1002/iid3.1251

**Published:** 2024-04-12

**Authors:** Bo Wang, Yongqiang Xiong, Ren Li, Jiewen Zhang, Shu Zhang

**Affiliations:** ^1^ Department of Geriatric Digestive Surgery the Second Affiliated Hospital of Xi'an Jiaotong University Xi'an China; ^2^ Experimental Teaching Center for Clinical Skills the Second Affiliated Hospital of Xi'an Jiaotong University Xi'an China

**Keywords:** immune cells, immunodeficiency, Mendelian randomization, single‐nucleotide polymorphism, telomere length

## Abstract

**Background:**

For a long time, the prevailing viewpoint suggests that shorter telomere contribute to chromosomal instability, which is a shared characteristic of both aging and cancer. The newest research presented that T cell immune deficiency rather than chromosome instability predisposes patients with short telomere syndromes to some cancers. However, the relationship between genetically determined telomere length (TL) and immune cells remains unclear.

**Methods:**

The two‐sample Mendelian randomization analysis was conducted to elucidate the potential causal relationship. The genetic data of TL and immune cells were obtained from the Genome‐Wide Association Study. The inverse variance weighted (IVW) method was used to estimate the effects primarily and another four methods were as a supplement. Sensitivity analysis was used to test the results.

**Results:**

The IVW method showed a significant correlation between TL and the percentage of T cells in lymphocytes (odds ratio [OR]: 1.222, 95% confidence interval [CI]: 1.014–1.472, *p* = .035), indicating that shorter TL significantly increases the risk of low T cell percentage. Further analysis of T cell subsets indicated that shorter TL may primarily lead to a lower percentage of Natural Killer T cells (OR: 1.574, 95% CI: 1.281–1.935, *p* < .001). Analysis of B cell subsets revealed that shorter TL may be associated with a higher percentage of Naive‐mature B cells, and a lower percentage of Memory B cells. And the sensitivity analysis indicated the validity and robustness of our findings.

**Conclusion:**

In summary, our findings suggest that shorter TL may be associated with a decline in the percentage of T cell, as well as impediments in the differentiation of B cell, consequently leading to the onset of immunosenescence and immunodeficiency. The relevant mechanisms and potential therapeutic avenues still need further investigation.

## Abstract (highlights)

Shorter telomere length may be associated with a decline in the percentage of T cell, as well as impediments in the differentiation of B cell, consequently leading to the onset of immunosenescence and immunodeficiency.

## 1 INTRODUCTION

Telomeres are repetitive DNA sequences, first described in the 1930s, located at the ends of chromosomes that play a crucial role in maintaining genome integrity and stability. Until the 1970s, Olovnikov posited that telomeres undergo incomplete replication during DNA replication, proposing a theory that correlates this phenomenon with the limitation of cell proliferation capacity and the concept of “duration of life” (marginotomy theory).^1^ At the same time he further suggested for germline cells and immortal cell populations like most cancer cell lines, an enzyme, telomerase, might be activated that would prevent the diminution of DNA termini at each cell division, thus protecting the information containing part of the genome. The DNA sequences shorten in dividing normal cells, and the enzyme responsible for maintaining telomere length (TL) constant in immortal cell populations, are respectively identified as telomeres and telomerase.^2^ Generally, TL tends to shorten with human physiological aging and is considered an important hallmark of aging.^3^, ^4^

Blood leukocytes are composed of multiple cell types that derive from myeloid and lymphoid lineages. Because the mature myeloid lineage cells are non‐dividing cells, whereas mature lymphocytes undergo extensive cell division in mediating their function, TL measured for leukocytes reflect sums or averages over cells with potentially very different replicative histories.^5^, ^6^ Many methods have been developed to measure TL, such as quantitative polymerase chain reaction (Q‐PCR), terminal restriction fragment (TRF) analysis and a variety of quantitative fluorescence in situ hybridization (Q‐FISH) methods, and so on.^7^ The Q‐PCR approach stands out for its simplicity and suitability, rendering it widely utilized in high‐throughput data analysis and large‐scale population studies. In this procedure, relative TL was determined by calculating the T/S ratio (T, the amplification of the telomere product; S, the amplification of a single copy gene), a value that is proportional to average TL.^8^

A study on systemic inflammatory diseases revealed that alterations in telomerase activity and TL were observed in various systemic immune‐mediated diseases and appeared to be associated with premature immune aging.^9^ One study also shows that telomeres and telomerase play crucial roles in T‐cell differentiation, aging, and diseases.^10^ At the same time, prospective studies and meta‐analysis have also found that shorter TL increases the incidence and mortality of cancer, especially in bladder, esophageal, gastric, head, and neck cancers.^11^, ^12^ The prevailing viewpoint suggests that shorter telomere contribute to chromosomal instability, which is a shared characteristic of both aging and cancer.^4^, ^13^ However, two articles recently presented a paradox at the intersection of aging and cancer biology, both short and long extremes of TL appear to mediate two distinct age‐associated disease phenotypes, illustrated in the predisposition to B‐cell and T‐cell lymphoproliferative or immunodeficiency, rather than chromosomal instability.^14^, ^15^ These findings led us to consider whether there is some causal relationship between TL and lymphocytes.

Mendelian randomization (MR) analysis is a genetic epidemiological approach aimed at investigating whether a specific exposure has a causal relationship with a particular outcome. MR utilizes genetic variations closely associated with the exposure as instrumental variables (IV) to infer the causal effect of the exposure on the specific outcome.^16^, ^17^ Due to Mendel's law of inheritance, which states that alleles are randomly allocated from parents to offspring during gamete formation, genetic variations are not influenced by conventional confounding factors. The temporal relationship between genetic variations and outcomes is therefore plausible. Thus, MR minimizes common sources of confounding and reverse causality biases commonly observed in observational studies, providing stronger evidence than observational studies.^18^, ^19^ In this study, we employed a two‐sample MR analysis to evaluate the relationship between TL and lymphocyte.

## 2 METHODS

### 2.1 Study design and data sources

This study utilized summary‐level data from published studies and databases and all genetic data can be obtained through summary data of Genome‐Wide Association Studies (GWAS), available at https://gwas.mrcieu.ac.uk/. Therefore, ethical approval from the institutional review board is not required for this research.

We conducted a two‐sample MR analysis to assess the causal relationship between TL and lymphocytes and their subsets. The summary GWAS results for TL were derived from 472,174 genetically well‐characterized adults in the UK Biobank (UKB). In this study, TL was quantified as the relative TL by Q‐PCR method, and the genetic variations were adjusted for age and sex.^8^, ^20^, ^21^ The GWAS data for lymphocytes were obtained from a genetic study focusing on multiple immunological traits in a European population of 7313 individuals, which identified complex genetic regulation of immune cells.^22^ Genetic data pertaining to myeloid‐derived cells were obtained from a separate GWAS conducted within a European population, aimed at elucidating the allelic spectrum of variation in blood cell traits and their associations with prevalent complex diseases.^23^ Further details regarding the GWAS summary data can be found in Table S1.

### 2.2 Genetic instrumental variable selection

**Figure 1:**
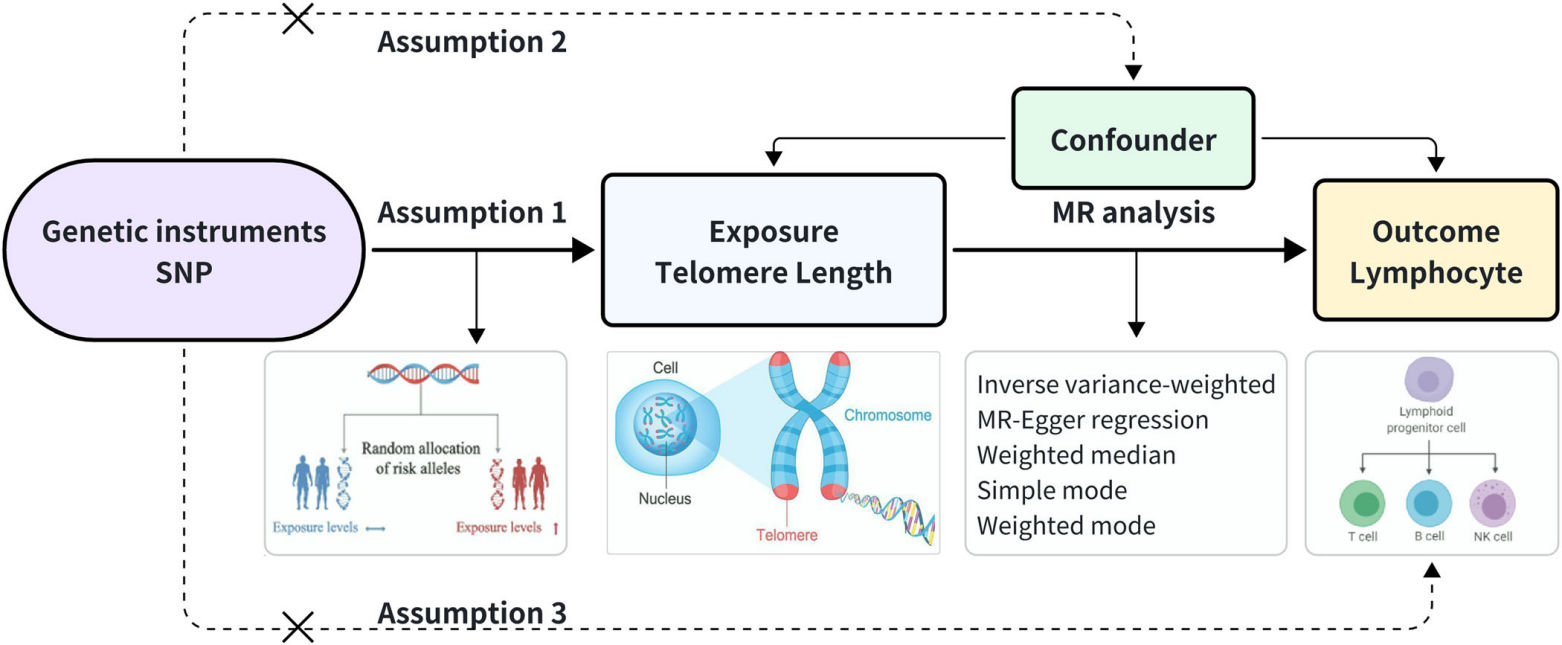
Schematic representation of the two‐sample Mendelian randomization analysis. Assumption 1 represents that genetic instrumental variables (IVs) are strongly associated with exposure and only affected the outcome through the exposure; assumption 2 represents that IVs are not associated with confounders; assumption 3 represents that IVs are not directly associated with the outcome.

Three fundamental conditions of MR analysis should be satisfied to ensure the validity of genetic variants as IVs. The MR assumptions were depicted in Figure 1 and listed as follows^1^: The genetic variations considered as IVs should be closely associated with the exposure,^2^ the genetic variations designated as IVs should not be associated with any confounding factors, and^3^ the genetic variations used as IVs should only influence the risk of the outcome through the exposure.^16^, ^17^ We performed rigorous filtering steps to control for single‐nucleotide polymorphism (SNP) quality in the merging of two distinct GWAS datasets. First, SNPs associated with the TL were selected using a genome‐wide significance threshold (*p* < 5 × 10^−8^). Second, SNPs with high overall linkage disequilibrium (LD, *R* ^2^ > 0.001 and 10,000 kb) were excluded. Third, SNPs with an *F*‐statistic less than 10 were omitted to ensure the strength of the genetic instruments. Additionally, after data harmonization, palindromic SNPs were excluded as they have intermediate allele frequencies.^18^, ^24^

### 2.3 MR analysis and sensitivity analysis

We primarily employed the inverse variance‐weighted (IVW) method to assess the impact of the TL on the percentage of different immune cells.^25^ Additionally, we utilized four supplementary analyses to confirm the results, including weighted mode, weighted median, simple mode, and MR‐Egger regression.^26^, ^27^, ^28^ After conducting the MR analysis, we performed sensitivity analyses including heterogeneity and pleiotropy assessment. Heterogeneity among SNP‐specific causal estimates was evaluated using meta‐analysis methods. Therefore, Cochran's *Q* statistic was calculated to measure heterogeneity between the estimated effects of individual TL‐associated SNPs on outcomes,^29^ the *p*‐value greater than 0.05 indicating a lack of heterogeneity. To investigate whether the TL‐associated SNPs exhibit pleiotropy, the MR‐Egger regression test was employed to identify potential pleiotropy, with a *p*‐value greater than 0.05 for the MR‐Egger intercept indicating a lack of horizontal pleiotropy.^28^ Additionally, Mendelian Randomization Pleiotropy RESidual Sum and Outlier (MR‐PRESSO) analysis was conducted to detect wide‐ranging horizontal pleiotropy in the causal relationship.^30^ Furthermore, leave‐one‐out analysis was employed to identify relevant SNPs that may influence the causal effects and identify potential outliers. The relationship was quantified by calculating odds ratios (OR) with 95% confidence intervals (CI). Scatter plots and funnel plots were generated to provide a clear visualization.

### 2.4 Statistical analysis

Statistical analysis and visualization were conducted using the “TwoSampleMR”^31^ and “MR‐PRESSO”^30^ packages in R version 4.2.2. All presented *p*‐values were two tailed, and *p*‐value less than .05 were considered statistically significant.

## 3 RESULTS

### 3.1 Selection of instrumental variables

According to the selection criteria, we included a total of 154 SNPs that are closely associated with Tl, with all of them exhibiting a significance level below *p* < 5 × 10^−8^. Additionally, we calculated the *F*‐statistic for each SNP, ranging from 29.9 to 1628.8, all of which exceeded 10. This indicates that these instrumental variables are unlikely to be influenced by instrument bias. The characteristics of these SNPs and the details of their association with TL, including *p*‐values, beta coefficients, standard errors, effect alleles, and *F*‐statistics, are presented in Table S2.

### 3.2 Results of MR analysis

**Figure 2:**
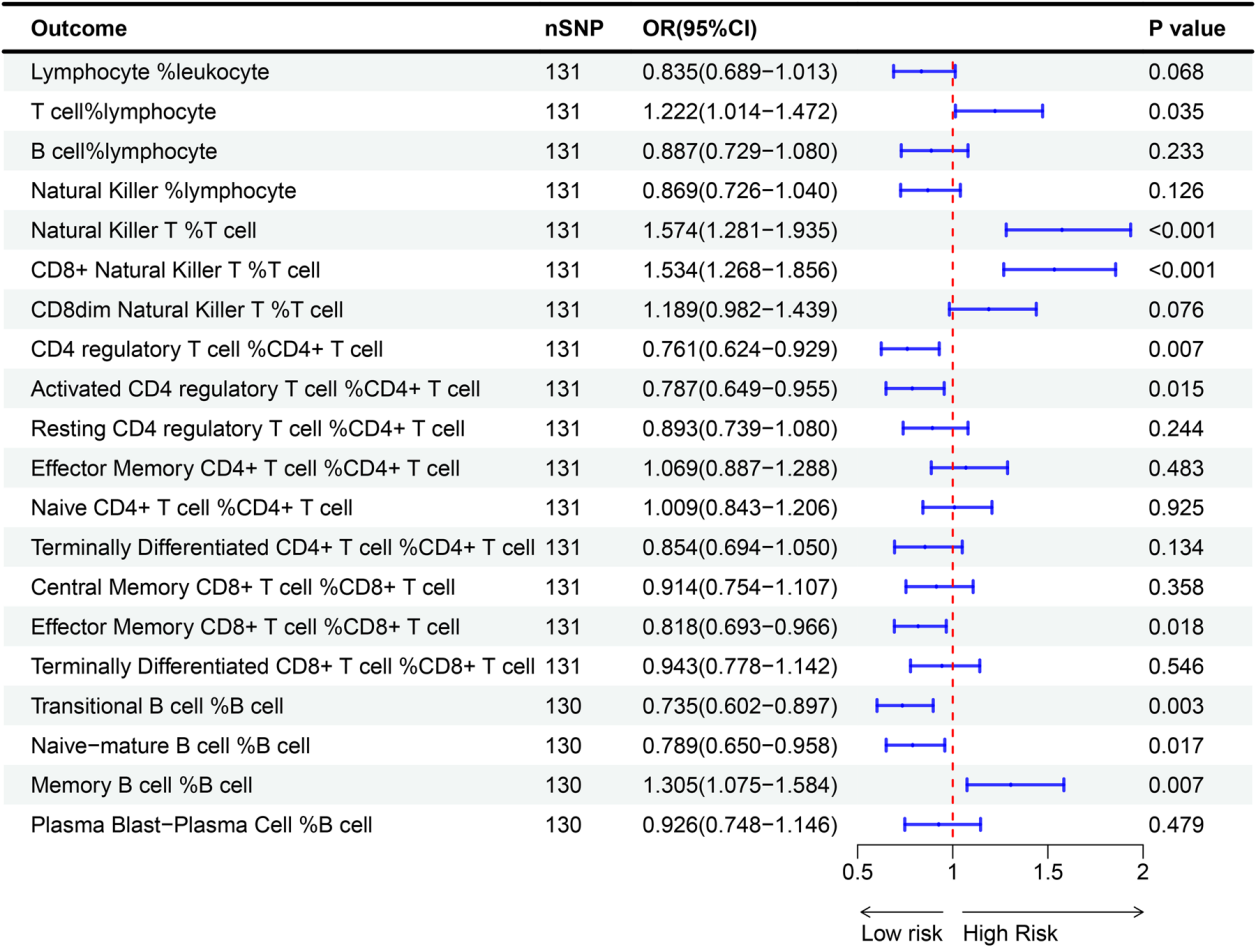
Forest plot of the association between genetically predicted telomere length and the percentage of immune cells. OR means a change in outcome risk associated with a 1‐standard deviation decrease in genetically determined telomere length. CI, confidence interval; IVW, inverse variance weighted; OR, odds ratio.

We analyzed primarily the causal relationship between TL and the percentage of immune cells using the inverse variance weighted (IVW) method, and the results are shown in Figure 2 and Table S3. Overall, there was no causal relationship between TL and the percentage of lymphocyte in leukocyte (OR: 0.835, 95% CI: 0.689–1.013, *p* = .068). Further results revealed a significant correlation between TL and the percentage of T cell in lymphocyte (OR: 1.222, 95% CI: 1.014–1.472, *p* = .035), indicating a causal relationship between shorter TL and lower T cell percentage. However, no significant association was observed between TL and the percentage of B cell (OR: 0.887, 95% CI: 0.729–1.080, *p* = .233) and Natural Killer cell (OR: 0.869, 95% CI: 0.726–1.040, *p* = .126).

Further analysis of T cell subsets indicated that shorter TL may lead to a lower percentage of Natural Killer T cells in T cells (OR: 1.574, 95% CI: 1.281–1.935, *p* < .001), particularly CD8+ Natural Killer T cell (OR: 1.534, 95% CI: 1.268–1.856, *p* < .001). However, no significant association was found between TL and CD8^dim^ Natural Killer T cell. Additionally, shorter TL was associated with a higher percentage of CD4 regulatory T cells in CD4^+^ T cells (OR: 0.761, 95% CI: 0.624–0.929, *p* = .007), especially Activated CD4 regulatory T cell (OR: 0.787, 95% CI: 0.649–0.955, *p* = .015), as well as a higher percentage of Effector Memory CD8^+^ T cell in CD8^+^ T cell (OR: 0.818, 95% CI: 0.693–0.966, *p* = .018). However, there do not appear to be causal relationships between TL and other CD4^+^ and CD8^+^ cells. Analysis of B cell subsets revealed that shorter TL may be associated with a higher percentage of Transitional B cell in B cells (OR: 0.735, 95% CI: 0.602–0.897, *p* = .003), Naive‐mature B cell in B cells (OR: 0.789, 95% CI: 0.650–0.958, *p* = .017), and a lower percentage of Memory B cell in B cells (OR: 1.305, 95% CI: 1.075–1.584, *p* = .007).

**Figure 3:**
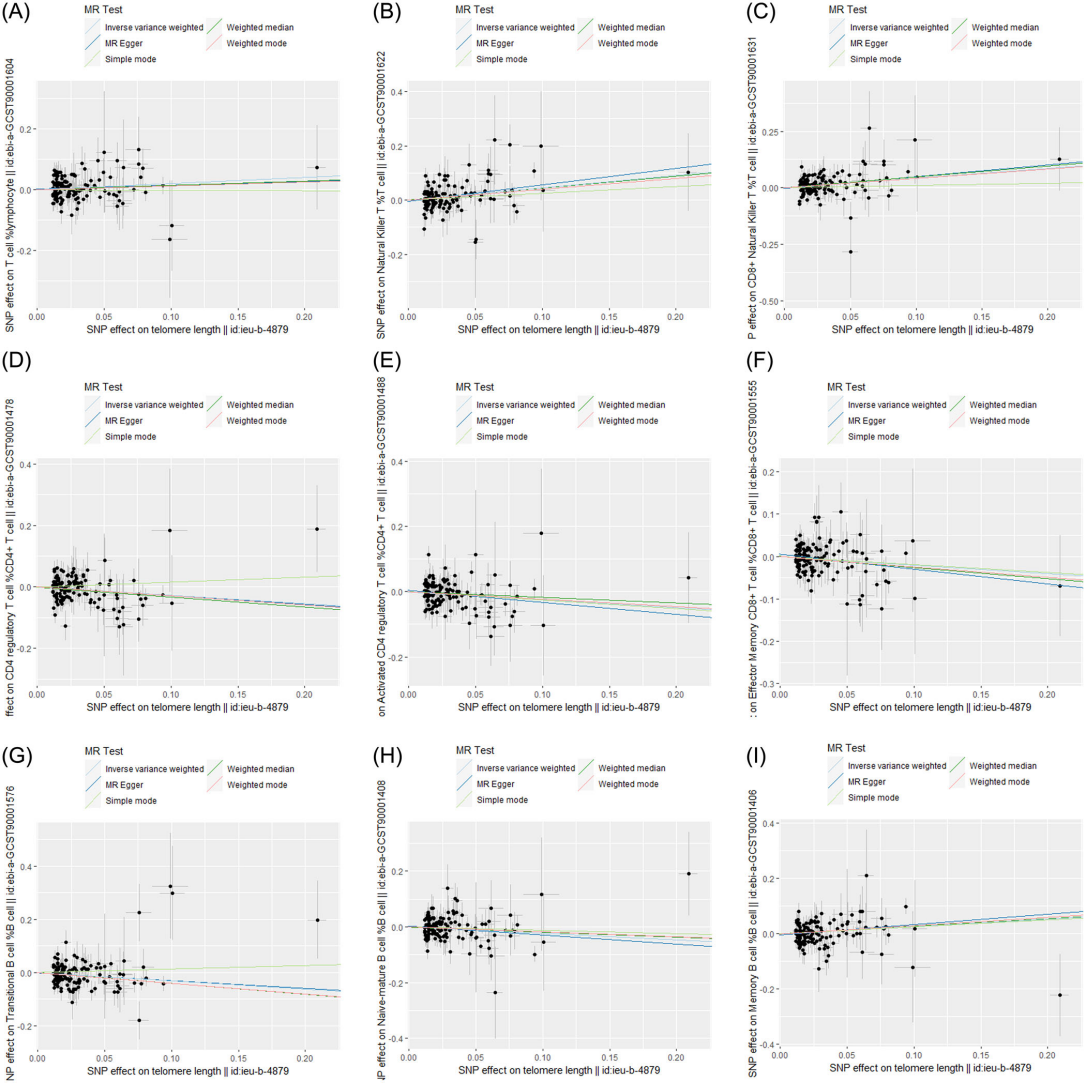
Scatter plots showing the causal effect of SNPs on telomere length against the effects on the quantity of immune cells. SNP, single nucleotide polymorphisms; MR, Mendelian randomization. (A–I) T cell % lymphocyte, natural killer T %T cell, CD8^+^ natural killer T %T cell, CD4 regulatory T cell % CD4^+^ T cell, Activated CD4 regulatory T cell %CD4^+^ T cell, effector memory CD8^+^ T cell %CD8^+^ T cell, transitional B cell %B cell, naive‐mature B cell %B cell, Memory B cell %B cell.

Scatter plots also clearly illustrate this relationship (Figure 3). The results of the four additional supplementary analyses, including weighted mode, weighted median, simple mode, and MR‐Egger regression, also support the same conclusion. Details of all results of the MR analysis are provided in Table S3.

### 3.3 Sensitivity analysis

For the stability of the results, we conducted the sensitivity analysis. The results of heterogeneity and pleiotropy analyses can be found in Table 1. Cochran's *Q* test conducted for both MR Egger and IVW indicated no evidence of heterogeneity among these SNPs (*p* > .05). Similarly, in the funnel plots of IVW and MR Egger, no significant heterogeneity between IVs was observed (Figure S1). Furthermore, as shown in Table 1, in the pleiotropy analysis, the MR Egger regression test did not reveal any evidence of directional pleiotropy. The results of MR‐PRESSO global test also indicated that there was no directional pleiotropy and that there were no outliers for any IVs. This suggests that the obtained results are unlikely to be influenced by any potential biases. Additionally, the leave‐one‐out test showed that the causal estimation was not influenced by single SNP (Figure S2). No matter which SNP was removed, it would exert no fundamental effect on the results, implying the stability of our two‐sample MR analysis.

**Table 1:** Heterogeneity and pleiotropy tests for instrumental variables.

Outcome	Method	nSNP	Cochran *Q*	*P*_Heterogeneity_	*P*_Pleiotropy_
T cell % lymphocyte	IVW	131	124	0.637	0.635^a^
	MR Egger	131	123	0.621	0.573^b^
Natural killer T %T cell	IVW	131	147	0.140	0.141^a^
	MR Egger	131	146	0.140	0.332^b^
CD8^+^ Natural killer T %T cell	IVW	131	132	0.445	0.420^a^
	MR Egger	131	131	0.430	0.520^b^
CD4 regulatory T cell %CD4^+^ T cell	IVW	131	134	0.393	0.408^a^
	MR Egger	131	134	0.369	0.938^b^
Activated CD4 regulatory T cell %CD4^+^ T cell	IVW	131	131	0.464	0.454^a^
	MR Egger	131	130	0.459	0.375^b^
Effector memory CD8^+^ T cell %CD8^+^ T cell	IVW	131	122	0.671	0.662^a^
	MR Egger	131	121	0.681	0.245^b^
Transitional B cell %B cell	IVW	130	141	0.222	0.221^a^
	MR Egger	130	141	0.204	0.911^b^
Naive‐mature B cell %B cell	IVW	130	126	0.564	0.569^a^
	MR Egger	130	125	0.547	0.560^b^
Memory B cell %B cell	IVW	130	125	0.572	0.546^a^
	MR Egger	130	125	0.562	0.449^b^

Abbreviations: IVW, inverse‐variance‐weighted; nSNP, number of single‐nucleotide polymorphism; MR‐PRESSO, MR pleiotropy residual sum and outlier; *P*_Heterogeneity_, *p*‐value of the Cochran's *Q* test for heterogeneity by IVW and MR Egger. *P*_Pleiotropy_, *p*‐value of the horizontal pleiotropy.

^a^ *p*‐Value of MR‐PRESSO global test.

^b^ *p*‐Value of the intercept from MR Egger regression analysis.

In addition, we also selected genetic data of myeloid‐derived cells from another study to perform MR analysis. The results of IVW, shown in Table S4, indicate a causal relationship between TL and the neutrophil percentage of white cells, that is, a shorter TL suggests a lower neutrophil percentage of white cells (OR: 1.133, 95% CI: 1.063–1.207, *p* < .001). Meanwhile, a shorter TL may also contribute to a higher percentage of eosinophil in white cells (OR: 0.876, 95% CI: 0.816–0.940, *p* < .001). However, there does not appear to be a significant causal relationship between TL and basophil percentage of white cells (OR: 1.005, 95% CI: 0.952–1.061, *p* = .865) and monocyte percentage of white cells (OR: 0.956, 95% CI: 0.865–0.1.058, *p* = .386). We also performed a sensitivity analysis and, unfortunately, the results suggest that there were significant heterogeneity (*P*_Heterogeneity_ < 0.05) between these SNPs by the MR Egger and IVW methods (Table S4). This may bias the estimation of the causal effect, making the results unreliable.

## 4 DISCUSSION

As medical technology continues to advance, the average human life expectancy has been on the rise, prompting increased attention to the phenomenon of aging within the medical community. A spectrum of age‐related conditions, encompassing malignancies, cardiovascular diseases, metabolic disorders, and degenerative ailments, has emerged as focal points of research interest.^20^, ^32^ Telomeres play a key role in the maintenance of genome integrity and stability. Previous studies have shown that telomeres cannot fully replicate during normal cell division and shortening occurs. In some germ cells or immortal cells, such as most cancer cells, they activate telomerase to maintain a constant TL.^1^, ^2^ Meanwhile, there is a strong link between shorter TL and the incidence and mortality of some cancers.^11^, ^12^ These all indicate that telomeres are the meta‐Hallmarks of aging and cancer and this role is exerted by affecting chromosomal stability.^4^, ^13^ However, a recent study from the Johns Hopkins University School of Medicine has overturned our previous understanding by revealing that T cell immune deficiency rather than chromosome instability predisposes patients with short telomere syndromes to some cancers.^14^ Simultaneously, previous studies have suggested that telomerase activity and TL may be altered in various systemic immuno mediated diseases and appear to be associated with premature immune aging.^9^ Moreover, TL as well as telomerase is also strongly associated with T cell differentiation and aging.^10^ These prompted us to explore the association between TL and immune cells, especially lymphocytes.

MR is a novel approach that utilizes genetic variation as instrumental variables to determine the impact of certain exposure on outcome. Genetic variation is essentially randomly inherited from parents to offspring at conception, and consequently, many factors that confound the association between the exposure and outcome cannot affect the genetic variants. Similarly, genetic variants are generally not influenced by the outcome and therefore, by reverse causation.^16^, ^18^ Thus, we employed a two‐sample MR method to study the association between TL and immune cells while reducing potential bias from confounding and reverse causation.

Our results indicate a clear association between TL and the percentage of immune cells and their subsets. Overall, there is no apparent causal relationship between TL and the percentage of lymphocytes in leukocytes. Further analysis reveals a correlation between TL and the percentage of T cells in lymphocytes; specifically, shorter TL is associated with a lower percentage of T cells in lymphocytes. Subsequent subset analysis suggests that this relationship is notably manifested in a lower percentage of Natural Killer T cell in T cells, particularly CD8^+^ Natural Killer T cells. Simultaneously, shorter TL may also lead to an increase in the proportion of CD4 regulatory T cells and Effector Memory CD8^+^ T cells. Changes in the proportions of these lymphocyte subsets typically signify the occurrence of immunosenescence and immunodeficiency.^13^, ^33^, ^34^, ^35^, ^36^ In fact, theoretical biologist Olovnikov, following his groundbreaking work, a theory of marginotomy, discussed the potential outcome of thymic degeneration due to telomere shortening leading to the loss of T cell proliferative capacity.^1^, ^2^, ^10^ Recent animal experiments have also demonstrated that a mouse model with shortened telomeres results in hematopoietic defects, including reduced spleen size, decreased follicle numbers, decreased lymphocyte counts, and impaired lymphocyte proliferation.^37^ Additionally, a cohort study on patients with short telomere syndrome directly indicates severe depletion of T cells in carriers of telomerase mutations, suggesting this may be due to T cell autonomous apoptosis defects caused by short TL.^14^, ^38^ Concurrently, short telomeres may limit TCR diversity, leading to T cell differentiation disorders and immune deficiencies.^38^ Our MR analysis further supports this conclusion, demonstrating the occurrence of T cell immune defects associated with short TL after controlling for various confounding biases.

TL appears to lack a causal relationship with the overall percentage of B cell in lymphocytes. However, further subgroup analysis reveals that shorter TL typically leads to a decrease in the percentage of Memory B cell in B cells, accompanied by an increase in the percentage of Transitional B cell and Naive‐mature B cell in B cells. This suggests a potential hindrance in the proliferation and differentiation of B cells. It is well known that the maturation of B cells involves a series of differentiation processes, including immature B cells, transitional B cells, naive B cells, memory B cells, and plasma cells.^39^, ^40^ Previous studies have suggested that short TL may impact the proliferative capacity of B cell.^41^ Simultaneously, research on telomere dysfunction indicates that TL influences the proliferation and differentiation of B cells.^42^ These findings, in conjunction with our results, indicate that shorter TL can lead to differentiation barriers in B cells, resulting in adaptive immune deficiencies.

Unfortunately, in the study conducted by Orrù V, detailed genetic data regarding the myeloid derived cells were not available.^22^ Consequently, an alternative data set from another study was employed for the analysis of the relationship between TL and myeloid derived cells.^23^ The results suggest that a shorter TL may be associated with a lower percentage of neutrophil and a higher percentage of eosinophil of white cells. However, these findings did not withstand heterogeneity testing, indicating the potential unreliability of our conclusions. This discrepancy may be attributed to variations in analytical platforms, experimental conditions, and population selection between the two studies. Indeed, prior research has postulated that TL in progenitor cells sets an upper limit for the proliferative capacity of lineage descendants during differentiation within the myeloid lineage, including granulocyte and monocyte. TL in differentiating myeloid populations is correlated with their anticipated replication history.^37^ In contrast, lymphocyte lineages exhibit a more intricate interplay between telomerase activity and TL dynamics; for instance, antigenic stimuli can induce telomerase activation and telomere elongation in these cells.^10^, ^43^ This prompts us to focus our attention on the relationship between TL and lymphocyte rather than myeloid derived cell in our investigation.

The strength of our study resides in its innovative utilization of a two‐sample MR methodology to evaluate the influence of TL on immune cells. We meticulously selected instrumental variables based on rigorous criteria and applied diverse MR techniques for causal inference. Additionally, sensitivity analyses revealed no notable heterogeneity or evidence of horizontal pleiotropy, thereby affirming the credibility of our study outcomes. Our research establishes a causal relationship between genetically determined TL and immune cells, specifically indicating that shorter TL is associated with a decline of T cell percentage, as well as disruptions in B cell differentiation, ultimately leading to the onset of immunosenescence and immune defects.

Nevertheless, our study has certain limitations. First, our data set lacks individual‐level data related to specific factors, such as gender, the presence of diseases that may influence lymphocytes, restricting further analyses. Additionally, our study population exclusively comprises individuals of European descent, raising uncertainty about the generalizability of our findings to other populations. Second, the genetic data concerning myeloid and lymphoid cell sources are derived from separate studies, limiting our ability to conduct a more integrated analysis. Third, it is crucial to note that biological traits are influenced by a combination of genetic and environmental factors. Our study results can only partially elucidate the impact of TL on immune cells.

## 5 CONCLUSION

In summary, our findings suggest that shorter TL may be associated with a decline in the percentage of T cell, as well as impediments in the differentiation of B cell, consequently leading to the onset of immunosenescence and immunodeficiency. The relevant mechanisms and potential therapeutic avenues still need further investigation.

## AUTHOR CONTRIBUTIONS

Bo Wang and Shu Zhang designed the study. Bo Wang wrote the original draft. Yongqiang Xiong, Ren Li, and Jiewen Zhang together with Bo Wang and Shu Zhang analyzed the results and further revised the manuscript. All authors have read and approved the manuscript.

## CONFLICT OF INTEREST STATEMENT

The authors declare no conflict of interest.

## Supporting information

Supporting information.

Supporting information.

Supporting information.

## Data Availability

The datasets generated during and/or analyzed during the current study are available at https://gwas.mrcieu.ac.uk/. Further inquiries can be directed to the corresponding author.
