# Supplementary figures and images for: Shorter telomere length increases the risk of lymphocyte immunodeficiency: A Mendelian randomization study
Source: Immun Inflamm Dis. 2024 Apr 12;12(4):e1251. doi: 10.1002/iid3.1251 (PMC11010948; doi:10.1002/iid3.1251)

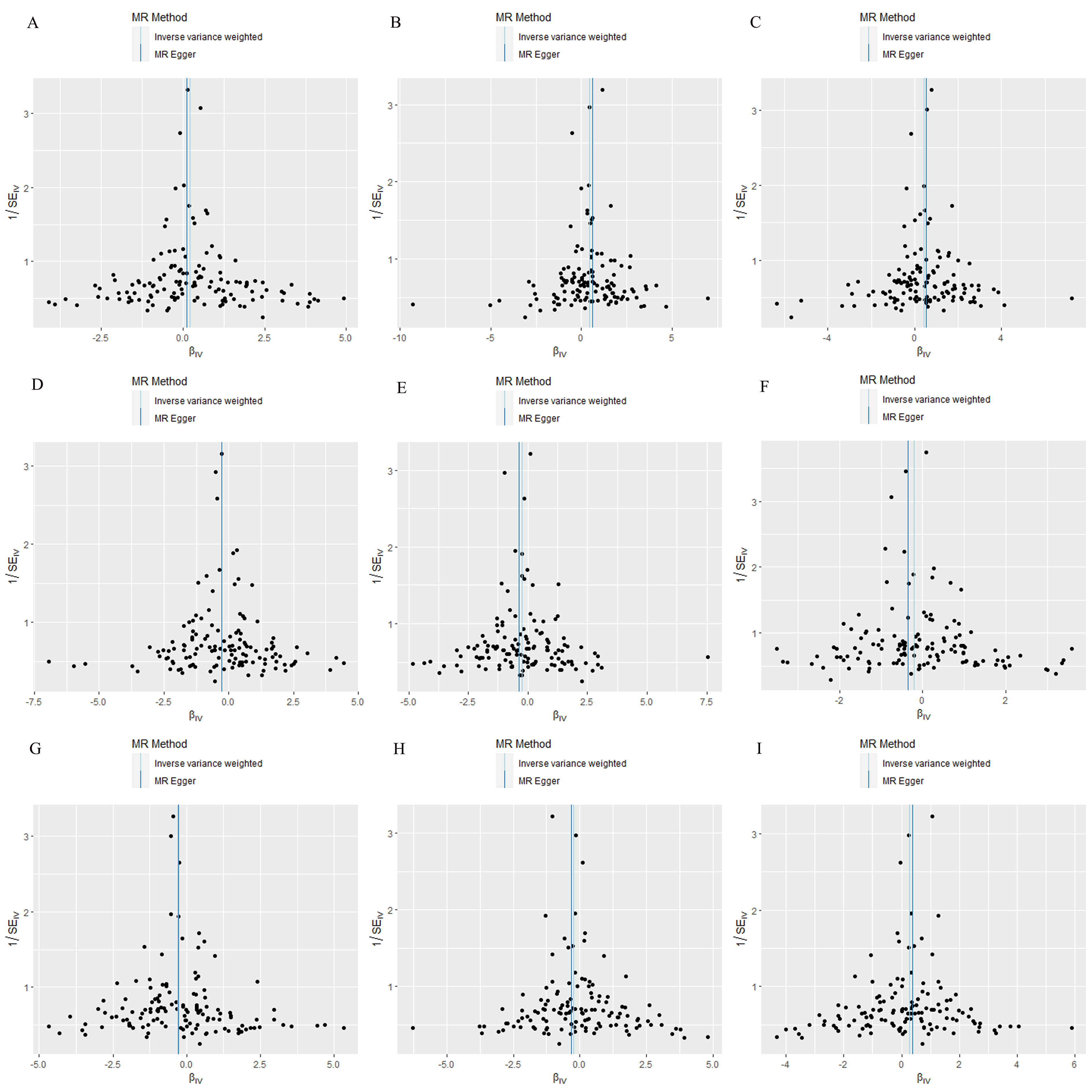

Supplement: Supplementary file 2 — Supporting information. [file IID3-12-e1251-s003.tif]
